# Supplementary material for: Lineage overwhelms environmental conditions in determining rhizosphere bacterial community structure in a cosmopolitan invasive plant
Source: Nat Commun. 2017 Sep 5;8:433. doi: 10.1038/s41467-017-00626-0 (PMC5585233; doi:10.1038/s41467-017-00626-0)
Supplement: Supplementary file 1 — Supplementary Information [file 41467_2017_626_MOESM1_ESM.pdf]

Description of Supplementary Files

File Name: Supplementary Information
Description: Supplementary Tables and Supplementary Figures

File Name: Peer Review File

Supplementary Table 1| Population identification, latitude, longitude, and lineage of field collected *Phragmites australis* samples. At each location, multiple individuals were collected and DNA and RNA were extracted from at least five replicate individuals from each location. The lineages of the *Phragmites australis* individuals was confirmed by RFLP analysis. NWR= National Wildlife Refuge. Samples with an asterisk indicate field populations that were among those used in the common garden experiment. Note that one Introduced population was included in the common garden experiment that was not a part of the original field survey.

Sample	Population	Latitude	Longitude	Collection Date	Lineage
BCI	Bonnet Carré Spillway, LA	30.0575	-90.372038	9/12/2015	Gulf
BICM*	Block Island, RI	41.17927	-71.56795	11/12/2016	Introduced
BICN*	Block Island, RI	41.17937	-71.56816	11/12/2016	Native
CA1	San Diego River, CA	32.76144	-117.2056	7/18/2015	Introduced
CA2	Salinas River, CA	35.49934	-120.65144	7/20/2015	Introduced
CA3	Palo Alto, CA	37.4348	-122.10126	7/21/2015	Introduced
CA4	Suisun, CA	38.0828	-122.10609	7/22/2015	Introduced
CRI	Creole 1, LA	29.83607	-93.11091	9/26/2015	Gulf
CT	Pettipaug Yacht Club, CT	41.36502	-72.38143	11/14/2015	Native
FONT1	Fontainebleau State Park, LA	30.337123	-90.04724	9/12/2015	Gulf
FPM*	Falmouth, MA	41.58692	-70.63737	12/14/2015	Introduced
FPN*	Falmouth, MA	41.58689	-70.63726	12/14/2015	Native
GBM*	Great Bay, NH	43.053978	-70.89545	11/1/2015	Introduced
NH*	Great Bay, NH	43.05581	-70.89943	7/3/2015	Native
OR	Port Oxford, OR	42.75671	-124.50052	7/25/2015	Native
PONT1	Pontchartrain 1, LA	30.30017	-90.404355	9/12/2015	Gulf
PONT2	Pontchartrain 2, LA	30.340644	-90.4123	9/12/2015	Gulf
RCM*	Rachel Carson NWR, ME	43.315988	-70.566463	10/31/2015	Introduced
RCN*	Rachel Carson NWR, ME	43.315988	-70.566463	10/31/2015	Native
RRN*	Rappahannock River, VA	37.91706	-76.85815	7/7/2015	Native
WA	Columbia River, WA	45.73225	-120.53137	7/27/2015	Native

Supplementary Table 2| Linear regression statistics for the distance similarity relationships described in Fig. 1B. Bold lines indicate a significant regression at $p < 0.05$.

	Slope	Intercept	df	*F* statistic	*p*-value	R^2
Within Gulf	$-1.8x10^{-4}$	0.55	8	2.63	0.14	0.25
**Within Introduced**	**$-1.55x10^{-5}$**	**0.26**	**16**	**5.94**	**0.022**	**0.19**
Within Native	$-2.79x10^{-6}$	0.55	26	0.74	0.39	0.03
**Between Introduced and Gulf**	**$-1.02x10^{-4}$**	**0.40**	**43**	**65.8**	**<0.01**	**0.59**
Between Introduced and Native	$-3.03x10^{-6}$	0.14	63	1.30	0.25	0.02
**Between Gulf and Native**	**$-1.57x10^{-5}$**	**0.42**	**38**	**5.20**	**0.02**	**0.12**

Supplementary Table 3| Kruskal-Wallis output for field samples using the Benjamini-Hochberg (BH) procedure to control for false discovery. Numbers are mean relative abundance for each lineage. Significance is defined with a BH corrected $p<0.01$.

Class	Order	Gulf	Native	Introduced	*F*	BH *p*
Field 16S rRNA gene						
Alphaproteobacteria	Rhizobiales	0.228	0.182	0.172	47.22	1.90E-07
Deltaproteobacteria	Myxococcales	0.067	0.273	0.084	46.24	3.03E-07
Gammaproteobacteria	Xanthomonadales	0.088	0.026	0.106	46.11	3.20E-07
Actinobacteria	Actinomycetales	0.073	0.031	0.058	43.42	1.15E-06
Betaproteobacteria	Burkholderiales	0.033	0.033	0.004	43.03	1.37E-06
Alphaproteobacteria	Rhodospirillales	0.027	0.041	0.026	42.43	1.82E-06
Acidobacteria-6	iii1-15	0.025	0.013	0.025	42.12	2.09E-06
Solibacteres	Solibacterales	0.018	0.023	0.024	41.79	2.44E-06
Deltaproteobacteria	Syntrophobacterales	0.002	0.004	0.005	41.24	3.15E-06
Pedosphaerae	Pedosphaerales	0.006	0.024	0.013	41.07	3.38E-06
Saprospirae	Saprospirales	0.037	0.013	0.051	40.89	3.68E-06
Alphaproteobacteria	Ellin329	0.039	0.015	0.031	40.47	4.46E-06
Deltaproteobacteria	Desulfobacterales	0	0	0.046	40.39	4.59E-06
Alphaproteobacteria	Rhodobacterales	0.018	0.005	0.029	39.57	6.75E-06
Cytophagia	Cytophagales	0.024	0.008	0.039	39.49	6.92E-06
Thermoleophilia	Solirubrobacterales	0.025	0.024	0.013	39.49	6.92E-06
Alphaproteobacteria	Unclass_Alpha.	0.015	0.009	0.011	39.41	7.10E-06
Acidobacteriia	Acidobacteriales	0.022	0.004	0.016	39.41	7.10E-06
Deltaproteobacteria	Caulobacterales	0.011	0.03	0.005	39.35	7.22E-06
Field 16S rRNA						
Alphaproteobacteria	Rhizobiales	0.142	0.086	0.146	69.88	7.11E-12
Deltaproteobacteria	Myxococcales	0.051	0.065	0.1	67.77	1.96E-11
Gammaproteobacteria	Xanthomonadales	0.013	0.006	0.019	66.67	3.28E-11

Actinobacteria	Actinomycetales	0.003	0.021	0.034	66.19	4.08E-11
Betaproteobacteria	Burkholderiales	0.043	0.021	0.05	64.07	1.13E-10
Alphaproteobacteria	Rhodospirillales	0.022	0.036	0.035	63.99	1.16E-10
Acidobacteria-6	iii1-15	0.029	0.065	0.034	63.89	1.20E-10
Solibacteres	Solibacterales	0.027	0.013	0.057	63.77	1.21E-10
Deltaproteobacteria	Syntrophobacterales	0.042	0.049	0.035	63.77	1.21E-10
Pedosphaerae	Pedosphaerales	0.055	0.006	0.024	63.76	1.21E-10
Saprospirae	Saprospirales	0.003	0.003	0.007	63.76	1.21E-10
Alphaproteobacteria	Ellin329	0.003	0.002	0.01	63.54	1.33E-10
Deltaproteobacteria	Desulfobacterales	0.073	0.062	0.013	63.23	1.53E-10
Alphaproteobacteria	Rhodobacterales	0.023	0.019	0.006	62.41	2.24E-10
Cytophagia	Cytophagales	0.003	0.003	0.001	59.76	7.98E-10
Thermoleophilia	Solirubrobacterales	0.002	0.002	0.009	58.12	1.73E-09
Alphaproteobacteria	Unclass. Alpha.	0.016	0.031	0.011	58.10	1.73E-09
Acidobacteriia	Acidobacteriales	0.007	0.001	0.02	57.81	1.97E-09
Deltaproteobacteria	Caulobacterales	0.001	0.002	0.013	57.08	2.78E-09

Supplementary Table 4| Kruskal-Wallis output for greenhouse samples using the Benjamini-Hochberg (BH) procedure to control for false discovery. Numbers are mean relative abundance for each lineage. Significance is defined with a BH corrected $p < 0.01$.

Class	Order	Gulf	Native	Introduced	*F*	BH *p*
Greenhouse 16S rRNA gene						
Alphaproteobacteria	Rhizobiales	0.238	0.201	0.104	32.114	2.20E-05
Deltaproteobacteria	Myxococcales	0.061	0.196	0.064	32.049	2.24E-05
Gammaproteobacteria	Xanthomonadales	0.086	0.041	0.045	31.733	2.58E-05
Actinobacteria	Actinomycetales	0.063	0.048	0.033	31.194	3.27E-05
Betaproteobacteria	Burkholderiales	0.038	0.051	0.027	31.191	3.27E-05
Alphaproteobacteria	Rhodospirillales	0.029	0.042	0.032	31.000	3.54E-05
Acidobacteria-6	iii1-15	0.027	0.014	0.039	30.800	3.85E-05
Solibacteres	Solibacterales	0.019	0.020	0.010	30.766	3.86E-05
Deltaproteobacteria	Syntrophobacterales	0.003	0.006	0.054	30.665	4.00E-05
Pedosphaerae	Pedosphaerales	0.009	0.022	0.026	30.570	4.05E-05
Saprospirae	Saprospirales	0.041	0.017	0.013	30.560	4.05E-05
Alphaproteobacteria	Ellin329	0.038	0.018	0.015	30.552	4.05E-05
Deltaproteobacteria	Desulfobacterales	0.000	0.000	0.023	30.387	4.33E-05
Alphaproteobacteria	Rhodobacterales	0.018	0.010	0.010	30.227	4.57E-05
Cytophagia	Cytophagales	0.024	0.008	0.008	30.225	4.57E-05
Thermoleophilia	Solirubrobacterales	0.020	0.025	0.011	30.106	4.66E-05
Alphaproteobacteria	Unclass. Alpha.	0.015	0.010	0.017	30.084	4.66E-05
Acidobacteriia	Acidobacteriales	0.020	0.004	0.018	30.082	4.66E-05
Deltaproteobacteria	Caulobacterales	0.011	0.026	0.007	30.078	4.66E-05
Greenhouse 16S rRNA						
Alphaproteobacteria	Rhizobiales	0.085	0.177	0.160	58.400	2.50E-09
Deltaproteobacteria	Myxococcales	0.040	0.081	0.267	56.970	2.56E-09
Gammaproteobacteria	Xanthomonadales	0.015	0.093	0.025	55.464	3.16E-09
Actinobacteria	Actinomycetales	0.009	0.065	0.031	55.162	3.16E-09

Betaproteobacteria	Burkholderiales	0.021	0.005	0.047	54.562	3.41E-09
Alphaproteobacteria	Rhodospirillales	0.023	0.029	0.038	49.773	3.12E-08
Acidobacteria-6	iii1-15	0.009	0.027	0.013	46.821	1.17E-07
Solibacteres	Solibacterales	0.022	0.027	0.032	43.354	5.79E-07
Deltaproteobacteria	Syntrophobacterales	0.008	0.007	0.006	42.817	6.73E-07
Pedosphaerae	Pedosphaerales	0.203	0.009	0.021	42.429	7.00E-07
Saprospirae	Saprospirales	0.006	0.053	0.013	42.338	7.00E-07
Alphaproteobacteria	Ellin329	0.007	0.027	0.012	42.150	7.05E-07
Deltaproteobacteria	Desulfobacterales	0.054	0.000	0.000	41.835	7.54E-07
Alphaproteobacteria	Rhodobacterales	0.026	0.028	0.004	41.708	7.54E-07
Cytophagia	Cytophagales	0.009	0.023	0.007	41.372	8.32E-07
Thermoleophilia	Solirubrobacterales	0.004	0.014	0.021	41.101	8.93E-07
Alphaproteobacteria	Unclass. Alpha.	0.011	0.014	0.007	40.543	1.06E-06
Acidobacteriia	Acidobacteriales	0.001	0.015	0.005	40.526	1.06E-06
Deltaproteobacteria	Caulobacterales	0.003	0.004	0.028	40.281	1.08E-06

Supplementary Table 5| Chi Squared likelihood ratio test statistics and significance values for the predictors and responses included in the structural equation model. Significant values are identified in bold.

Response	Predictor	$\chi 2$	DF	*p-value*
Microbial richness	**Lineage**	**5.9969**	**2**	**0.0499**
Microbial activity	**Lineage**	**9.0062**	**2**	**0.0111**
	Microbial richness	3.5263	1	0.0604
Microbial metabolism	**Lineage**	**25.4982**	**2**	**<0.001**
	Microbial richness	1.0847	1	0.2977
Belowground gallic acid	Microbial activity	0.0693	1	0.7923
	Microbial richness	0.3814	1	0.5368
	Metabolism	1.1212	1	0.2897
	Lineage	2.995	2	0.2237
Belowground carbon	Microbial activity	0.0122	1	0.9119
	**Microbial richness**	**5.0849**	**1**	**0.0241**
	Microbial metabolism	1.7884	1	0.1811
	**Lineage**	**13.5056**	**2**	**0.0012**
	**Belowground gallic acid**	**8.394**	**1**	**0.0038**
	Belowground biomass	1.6024	1	0.2056
	Aboveground biomass	0.2436	1	0.6216
Belowground nitrogen	Microbial activity	7.00E-04	1	0.9782
	Microbial richness	2.0674	1	0.1505
	Microbial metabolism	0.2382	1	0.6255
	Lineage	2.1292	2	0.3449
	Belowground biomass	1.2279	1	0.2678
	Aboveground biomass	0.8379	1	0.36
Belowground biomass	Microbial activity	0.8182	1	0.3657
	Belowground gallic acid	0.3317	1	0.5647
	Microbial richness	0.2426	1	0.6223
	Microbial metabolism	0.3617	1	0.5476
	**Lineage**	**7.179**	**2**	**0.0276**
Aboveground biomass	Microbial activity	0.552	1	0.4575
	Belowground gallic acid	0.158	1	0.691
	Microbial richness	0.8664	1	0.352
	Microbial metabolism	1.0607	1	0.303
	**Lineage**	**11.5158**	**2**	**0.0032**

Supplementary Table 6| Coefficients with corresponding *z*-tests from structural equation model regression relationships (a) and covariances (b). Significant values are identified in bold.

	Response	Predictor	Estimate	Standard Error	*P*
(a)	**Microbial richness**	**Lineage: Gulf**	**2533.885**	**126.040**	**<0.001**
	**Microbial richness**	**Lineage: Introduced**	**2530.027**	**53.733**	**<0.001**
	**Microbial richness**	**Lineage: Native**	**2259.921**	**101.461**	**<0.001**
	**Microbial activity**	**Lineage: Gulf**	**0.691**	**0.034**	**<0.001**
	**Microbial activity**	**Lineage: Introduced**	**0.673**	**0.025**	**<0.001**
	**Microbial activity**	**Lineage: Native**	**0.744**	**0.029**	**<0.001**
	Microbial activity	Microbial richness	1.588E-05	0.000	0.073
	**Microbial metabolism**	**Lineage: Gulf**	**3192428.389**	**27651.291**	**<0.001**
	**Microbial metabolism**	**Lineage: Introduced**	**3090687.120**	**20153.309**	**<0.001**
	**Microbial metabolism**	**Lineage: Native**	**3148845.181**	**23377.450**	**<0.001**
	Microbial metabolism	Microbial richness	-7.147	7.056	0.315
	Belowground gallic acid	Lineage: Gulf	-2127.776	2519.773	0.415
	Belowground gallic acid	Lineage: Introduced	-2063.762	2439.308	0.414
	Belowground gallic acid	Lineage: Native	-1954.199	2482.242	0.446
	Belowground gallic acid	Microbial richness	0.048	0.082	0.556
	Belowground gallic acid	Microbial metabolism	0.001	0.001	0.314
	Belowground gallic acid	Microbial activity	-162.779	644.892	0.802
	**Belowground carbon**	**Lineage: Gulf**	**53.910**	**10.898**	**<0.001**
	**Belowground carbon**	**Lineage: Introduced**	**54.556**	**10.478**	**<0.001**
	**Belowground carbon**	**Lineage: Native**	**55.537**	**10.665**	**<0.001**
	**Belowground carbon**	**Microbial richness**	**0.001**	**0.000**	**0.039**
	Belowground carbon	Microbial metabolism	-4.267E-06	0.000	0.216
	Belowground carbon	Microbial activity	-0.278	2.685	0.918
	Belowground carbon	Aboveground biomass	0.105	0.228	0.646
	Belowground carbon	Belowground biomass	-0.188	0.159	0.241
	**Belowground carbon**	**Belowground gallic acid**	**0.001**	**0.001**	**0.009**
	Belowground nitrogen	Lineage: Gulf	4.945	5.828	0.413
	Belowground nitrogen	Lineage: Introduced	4.680	5.615	0.421
	Belowground nitrogen	Lineage: Native	4.551	5.720	0.442
	Belowground nitrogen	Microbial richness	-2.166E-04	0.000	0.180
	Belowground nitrogen	Microbial metabolism	-8.437E-07	0.000	0.647
	Belowground nitrogen	Microbial activity	-0.038	1.465	0.980
	Belowground nitrogen	Aboveground biomass	-0.092	0.106	0.391
	Belowground nitrogen	Belowground biomass	-0.080	0.077	0.300

	Belowground biomass	Lineage: Gulf	-3.398	11.757	0.778
	Belowground biomass	Lineage: Introduced	-2.101	11.379	0.857
	Belowground biomass	Lineage: Native	-2.993	11.580	0.800
	Belowground biomass	Microbial richness	-1.384E-04	0.000	0.641
	Belowground biomass	Microbial metabolism	2.143E-06	0.000	0.569
	Belowground biomass	Microbial activity	-2.599	3.019	0.393
	Belowground biomass	Belowground gallic acid	0.000	0.000	0.586
	Aboveground biomass	Lineage: Gulf	11.454	7.485	0.152
	Aboveground biomass	Lineage: Introduced	10.465	7.245	0.174
	Aboveground biomass	Lineage: Native	10.422	7.370	0.183
	Aboveground biomass	Microbial richness	-1.947E-04	0.000	0.380
	Aboveground biomass	Microbial metabolism	-2.327E-06	0.000	0.331
	Aboveground biomass	Microbial activity	-1.351	1.911	0.483
	Aboveground biomass	Belowground gallic acid	0.000	0.000	0.707
(b)	~~ Belowground biomass	~~ Aboveground biomass	0.569		<0.001

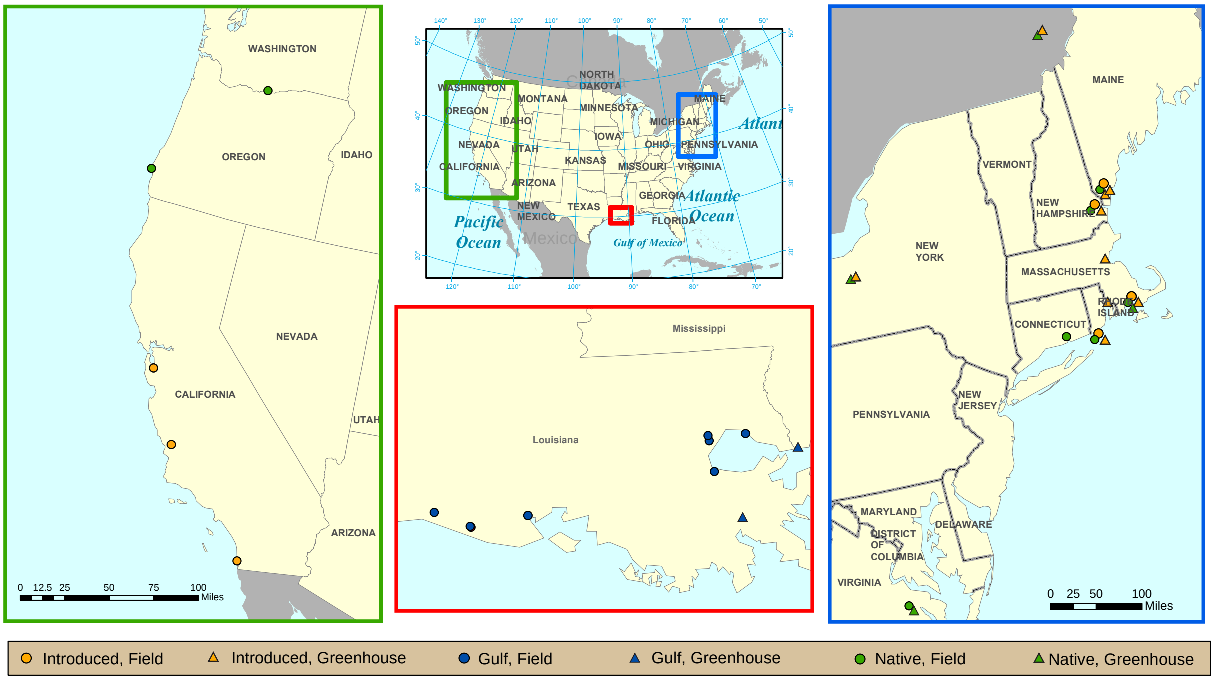

Supplementary Figure 1| Map of the locations of *Phragmites australis* populations. Points represent site locations used in both the field (circles) and greenhouse (triangles) experiments. Colors represent different lineages of *P. australis* (orange = Introduced, blue = Gulf, and green = Native). USA States Shapefile for constructing this map was published by Esri for ArcGIS® in 2012 using data from TomTom North America, Inc, the US Census Bureau, and the US Department of Agriculture National Agricultural Statistics Service.

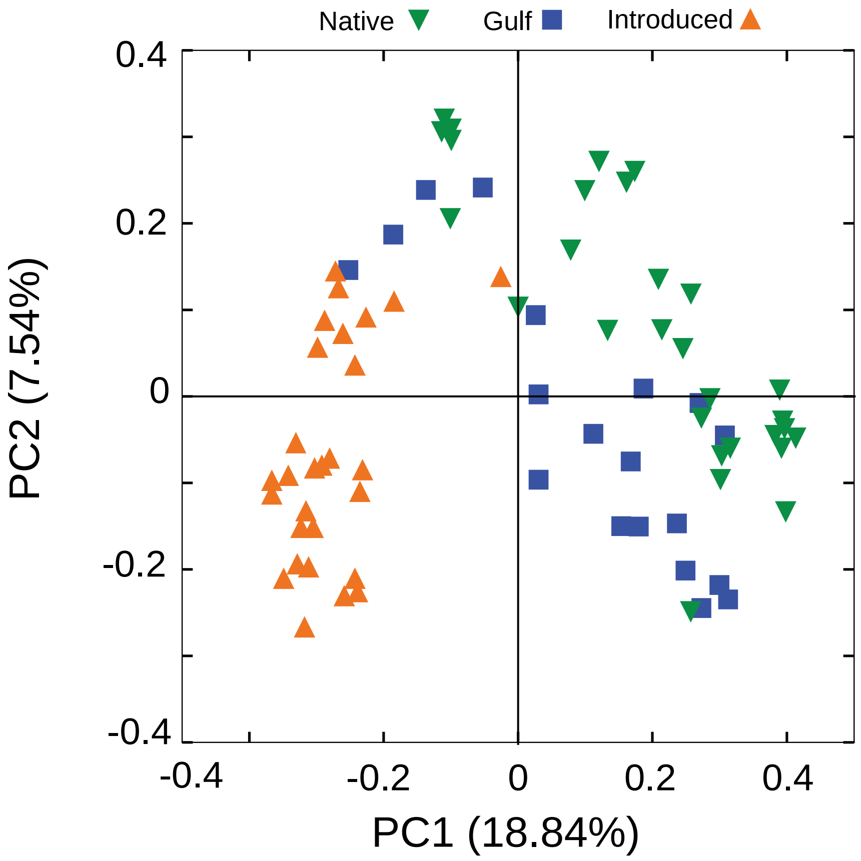

Supplementary Figure 2| Principal coordinates analysis of Bray-Curtis similarity of active microbes. Data represent the potentially active microbial communities associated with Native (green), Gulf (blue), and Introduced (orange) lineages of the common reed, *Phragmites australis*, collected from multiple locations around the United States (Supplementary Data Fig. 1). The three lineages have statistically different microbial communities in their associated rhizosphere soils (permutational multivariate analysis of variance, $F_{2,75} = 8.13$, $p < 0.001$) regardless of where in the country the samples were collected.

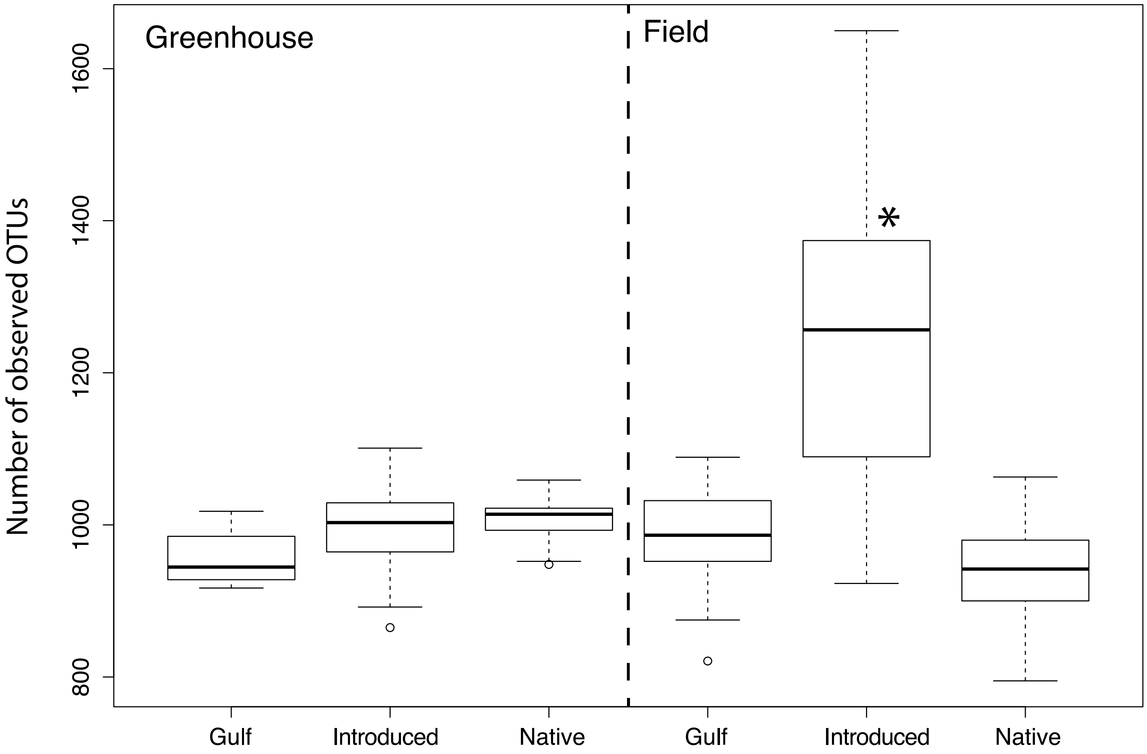

Supplementary Figure 3| Number of observed operational taxonomic units (OTUs) of rhizosphere microbe. Data are from both the greenhouse common garden experiment and samples collected in the field. Center bars are the median number of OTUs derived from data sets that were rarified to a sequencing depth of 5394 sequences per sample. The box indicates the 25th and 75th quartiles and whiskers are 1.5*interquartile range. The total number of microbial OTUs was significantly greater in the field collected rhizosphere soils from the Introduced lineage than in all other samples (ANOVA, $F_{5,206} = 56.5$, $p < 0.01$).
